# Supplementary material for: Photoswitchable Zirconium MOF for Light-Driven Hydrogen Storage
Source: Polymers (Basel). 2021 Nov 22;13(22):4052. doi: 10.3390/polym13224052 (PMC8618608; doi:10.3390/polym13224052)
Supplement: Supplementary file 1 [file polymers-13-04052-s001.zip › polymers-1478895-supplementary.pdf]

polymers

Supporting information

for

Photoswitchable Zirconium MOF for Light-Driven Hydrogen Storage

Vera V. Butova [1,*], Olga A. Burachevskaya [1], Vitaly A. Podshibyakin [2], Evgenii N. Shepelenko [3], Andrei A. Tereshchenko [1], Svetlana O. Shapovalova [1], Oleg I. Il'in [4], Vladimir A. Bren' [2] and Alexander V. Soldatov [1]

[1] The Smart Materials Research Institute, Southern Federal University, Sladkova 178/24, 344090 Rostov-on-Don, Russia; oburachevskaya@sfedu.ru (O.A.B.); antereshenko@sfedu.ru (A.A.T.); scherkasova@sfedu.ru (S.O.S.); soldatov@sfedu.ru (A.V.S.)
[2] Institute of Physical and Organic Chemistry, Southern Federal University, 344090 Rostov-on-Don, Russia; vpodshibakin@sfedu.ru (V.A.P.); vabren@sfedu.ru (V.A.B.)
[3] Federal Research Center the Southern Scientific Center of the Russian Academy of Sciences, 344006 Rostov-on-Don, Russia; e-shepelenko@mail.ru
[4] Institute of Nanotechnologies, Electronics and Equipment Engineering, Southern Federal University, Shevchenko 2, 347922 Taganrog, Russian; oiilin@sfedu.ru
* Correspondence: vbutova@sfedu.ru; Tel.: +7-863-297-51-28

Content

1. SEM 2
2. XRD 3
3. Nitrogen adsorption 4
4. TGA 5
5. FTIR 6
6. Stability 7

1. SEM

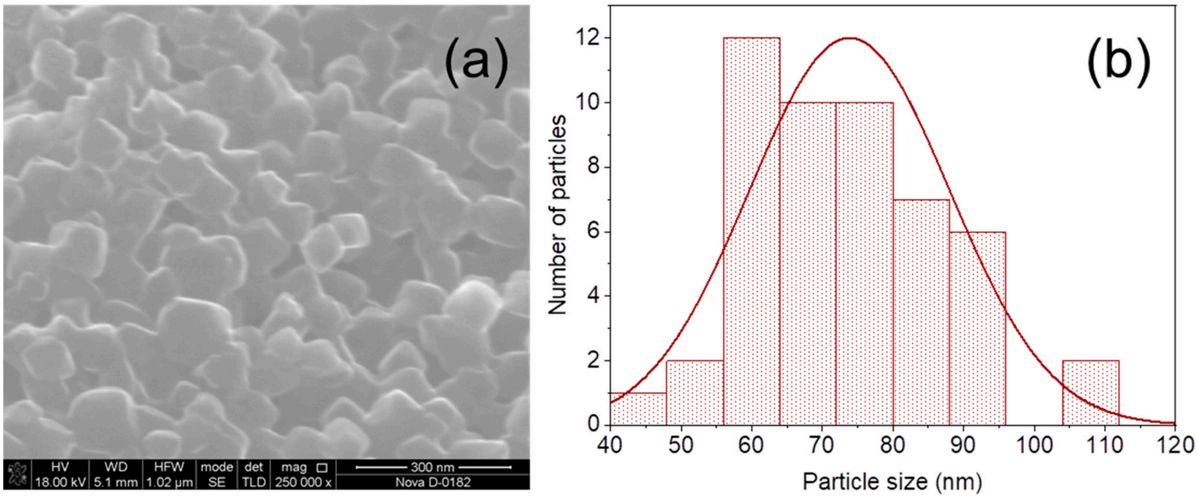

Figure S1. Representative SEM image of DAE-UiO-66 sample (**a**) and particle size distribution according to SEM data (**b**).

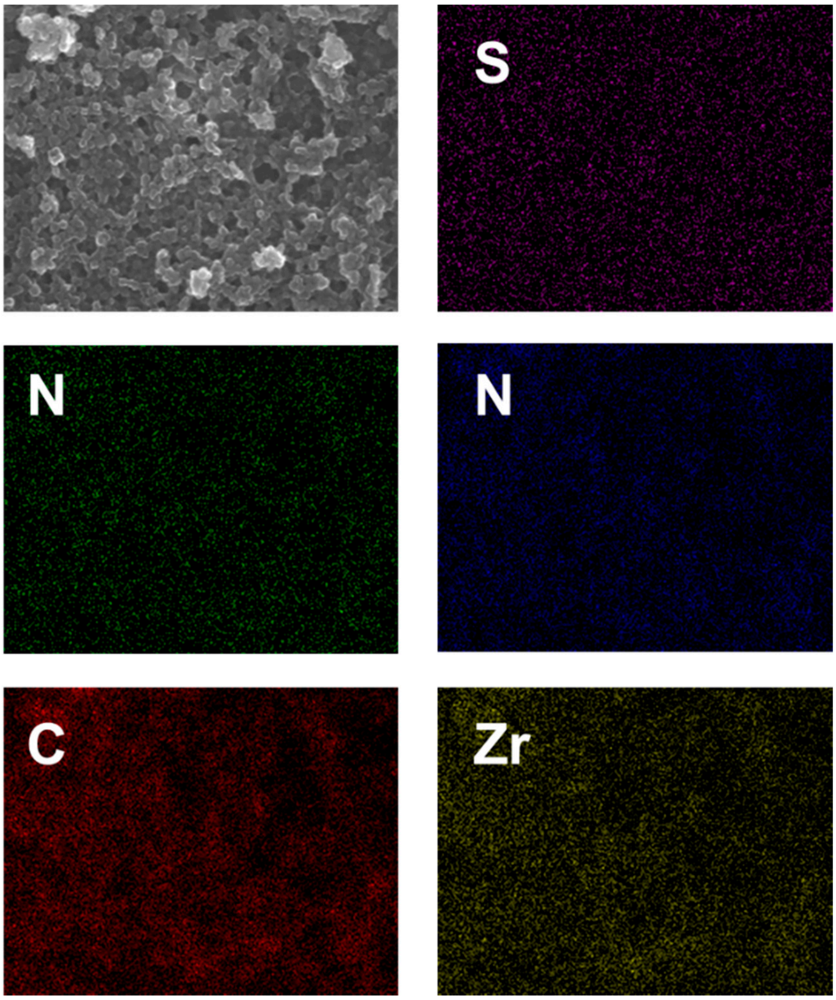

Figure S2. EDX-mapping of DAE-UiO-66 sample.

2. XRD

Table S1. Details of profile analysis.

Sample designation	Space group	Unit cell parameters		Agreement factors		
		a=b=c, Å	α=β=γ, °	R_P	wR_P	GOF
UiO-66-NH_2	Fm-3m	20.794(5)	90	0.1363	0.1812	3.53
DAE-UiO-66	(225)	20.836(6)	90	0.1146	0.1525	2.65

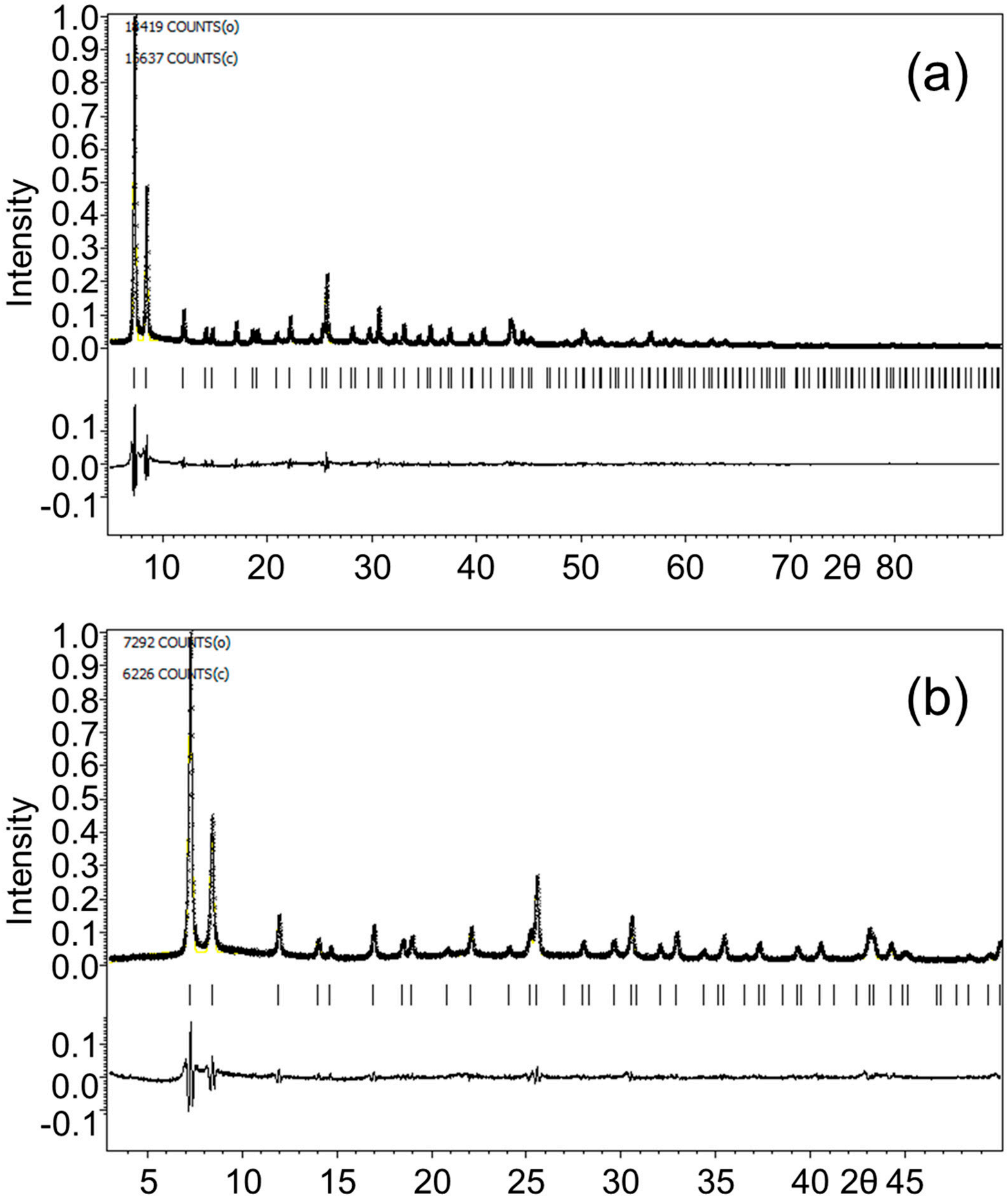

Figure S3. Experimental (black) and calculated (yellow) profiles of synthesized samples UiO-66-NH_2 (a) and DAE-UiO-66 (b) from fitting in Jana2006. Vertical lines indicate calculated pick positions. Difference plots are provided at the bottom of each picture.

Table S2. d-spacing and hkl indexes for observed reflections on XRD powder patterns for samples UiO-66-NH_2 and for DAE-UiO-66.

h	k	l	d spacing, Å		h	k	l	d spacing, Å	
			**UiO-66-NH_2**	**DAE-UiO-66**				**UiO-66-NH_2**	**DAE-UiO-66**
1	1	1	12.00731	12.02995	5	1	7	2.401462	2.40599
0	0	2	10.39864	10.41824	5	5	5	2.401462	2.40599
2	0	2	7.352946	7.366808	6	2	6	2.385611	2.390109
1	1	3	6.270614	6.282435	4	0	8	2.325206	2.32959
2	2	2	6.003655	6.014974	1	1	9	2.282797	2.287101
0	0	4	5.199318	5.20912	5	3	7	2.282797	2.287101
3	1	3	4.771221	4.780217	4	2	8	2.269168	2.273447
2	0	4	4.650411	4.659179	6	4	6	2.216997	2.221177
2	2	4	4.245225	4.253229	3	1	9	2.180146	2.184257
3	3	3	4.002437	4.009983	4	4	8	2.122613	2.126615
1	1	5	4.002437	4.009983	5	5	7	2.090204	2.094145
4	0	4	3.676473	3.683404	3	3	9	2.090204	2.094145
3	1	5	3.51538	3.522008	7	1	7	2.090204	2.094145
0	0	6	3.466212	3.472747	6	0	8	2.079727	2.083648
4	2	4	3.466212	3.472747	0	0	10	2.079727	2.083648
2	0	6	3.288337	3.294537	2	0	10	2.03934	2.043185
3	3	5	3.171554	3.177534	6	2	8	2.03934	2.043185
2	2	6	3.135307	3.141218	7	3	7	2.010548	2.014339
4	4	4	3.001828	3.007487	5	1	9	2.010548	2.014339
1	1	7	2.9122	2.917691	2	2	10	2.001218	2.004992
5	1	5	2.9122	2.917691	6	6	6	2.001218	2.004992
4	0	6	2.884063	2.8895	5	3	9	1.939356	1.943012
4	2	6	2.779152	2.784392	4	0	10	1.930978	1.934619
3	1	7	2.707574	2.712679	6	4	8	1.930978	1.934619
5	3	5	2.707574	2.712679	4	2	10	1.898522	1.902102
0	0	8	2.599659	2.60456	7	5	7	1.875227	1.878762
3	3	7	2.540791	2.545582	1	1	11	1.875227	1.878762
4	4	6	2.522039	2.526795	8	0	8	1.838236	1.841702
2	0	8	2.522039	2.526795	5	5	9	1.817066	1.820492
6	0	6	2.450982	2.455603	3	1	11	1.817066	1.820492
2	2	8	2.450982	2.455603	7	1	9	1.817066	1.820492

3. Nitrogen adsorption

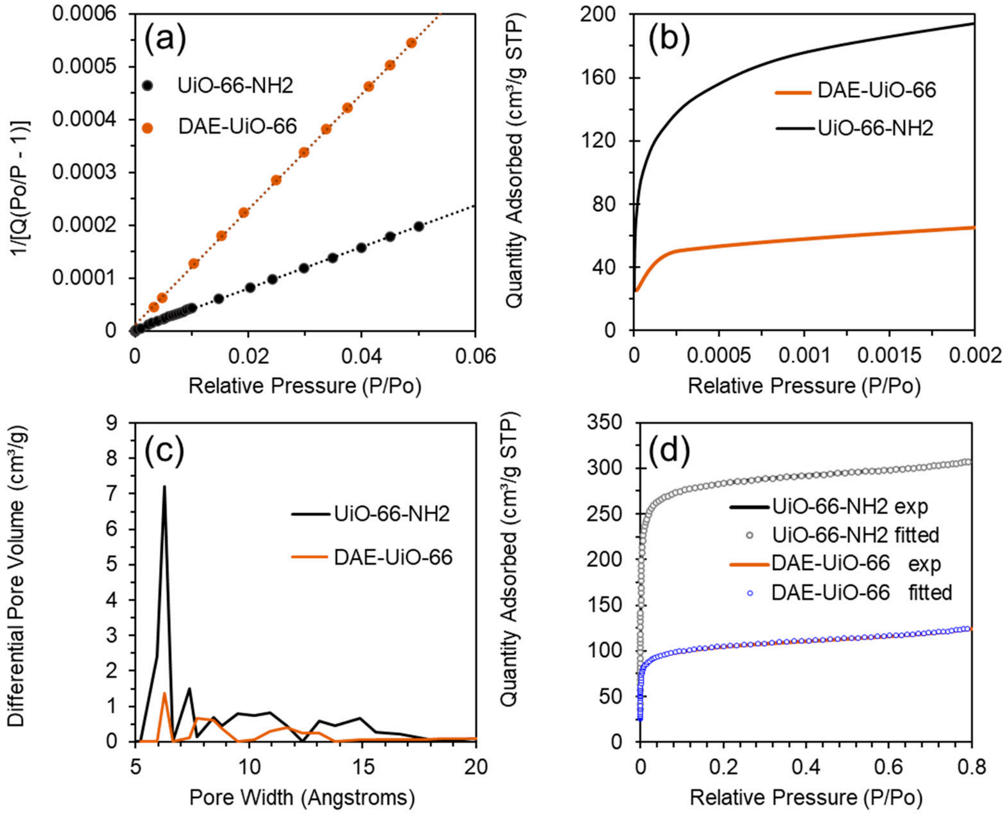

Figure S4. (**a**) BET Surface Area Plot. (**b**) Low-pressure region of nitrogen adsorption isotherms. (**c**) Pore size distribution. (**d**) Experimental data and model fit of nitrogen adsorption isotherms, which was used for pore-size distribution calculations.

Table S3. Details of calculations according to nitrogen adsorption isotherm of UiO-66-NH2 and DAE-UiO-66 samples.

	$UiO-66-NH_2$	DAE-UiO-66
SSA calculation		
Model	BET	BET
Surface Area, m^2/g	1111.3880 ± 4.7231	398.2522 ± 1.0596
Slope, g/cm^3 STP	0.003915 ± 0.000017	0.010918 ± 0.000029
Y-Intercept, g/cm^3 STP	0.000002 ± 0.000000	0.000012 ± 0.000001
C	1630.640045	889.771493
Qm, cm^3/g STP	255.3038	91.4850
Correlation Coefficient	0.9997561	0.9999646
Pore size distribution		
Method	2D-NLDFT	2D-NLDFT
Model	Tarazona, Cylindrical Pores	Tarazona, Cylindrical Pores
Standard Deviation of Fit, cm^3/g STP	0.87780	0.19886

4. TGA

The normalized curves were plotted according to recalculated values. For each sample, we have considered the chemical interaction of MOF with O2 from the air with the formation of gases and one solid product – ZrO_2. From one formula unit of UiO-66-NH_2 ($Zr_6O_8H_4(C_8H_5O_4N)_6$) six units of ZrO_2 were formed. So the solid residual in all cases is zirconium dioxide. So we used its amount as 100% and recalculated all weight losses in the following way: $WL_{norm}=WL_{exp}/Residual*100\%$.

Theoretical weight loss for UiO-66-NH_2:

$$WL_{theor} = \frac{M(MOF) - 6 \cdot M(ZrO_2)}{M(MOF)} \cdot 100\% = \frac{1718 - 739}{1718} \cdot 100\% = 57\%$$

Experimental weight loss for UiO-66-NH_2:

$$WL_{exp} = \frac{76 - 37}{76} \cdot 100\% = 51\ \%$$

Experimental weight loss for the UiO-66-NH_2 sample is lower than the theoretical one. We suppose it could be assigned to missing-linker defects in good agreement with previously reported data [1].

$$WL = \frac{M(MOF) - 6 \cdot M(ZrO_2)}{M(MOF)} \cdot 100\% = \frac{M(MOF) - 739}{M(MOF)} \cdot 100\% = 51\%$$

$$M\ (UiO\text{-}66\text{-}NH_2)_{exp} = 1519$$

Experimental weight loss for DAE-UiO-66:

$$WL_{exp} = \frac{86 - 32.3}{86} \cdot 100\% = 62.4\ \%$$

$$WL = \frac{M(MOF) - 6 \cdot M(ZrO_2)}{M(MOF)} \cdot 100\% = \frac{M(MOF) - 739}{M(MOF)} \cdot 100\% = 62.4\ \%$$

$$M\ (DAE\text{-}UiO\text{-}66)_{exp} = 1965$$

$$M[Zr_6O_4(OH)_4(C_8H_5O_4N)_{6-x}(C_{29}H_{23}O_7N_3S)_x] = 1565$$

$$M[Zr_6C_{48+23x}H_{34-3x}O_{32+3x}N_{6+2x}S_x] = 1565$$

$$x = 0.56$$

XRF: $M[Zr_6O_4(OH)_4(C_8H_5O_4N)_{5.2}(C_8H_6O_4NCl)_{0.3}(C_{29}H_{23}O_7N_3S)_{0.5}] = 1954.3$

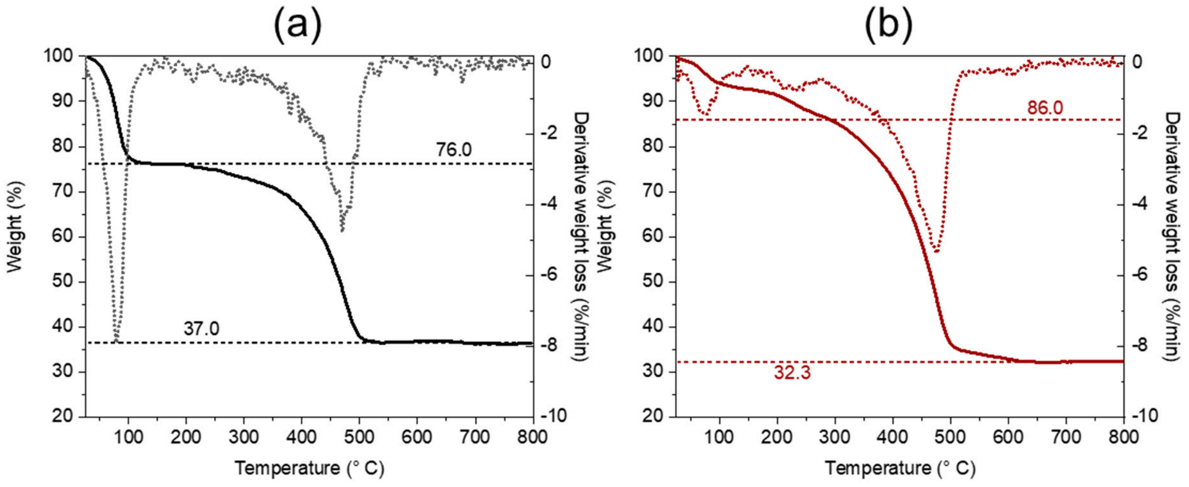

Figure S5. TGA (solid lines) and DTG (dotted lines) curves of samples UiO-66-NH_2 (**a**) and DAE-UiO-66 (**b**). Dashed lines represent exact values of weight losses, which were used for calculations.

5. FTIR

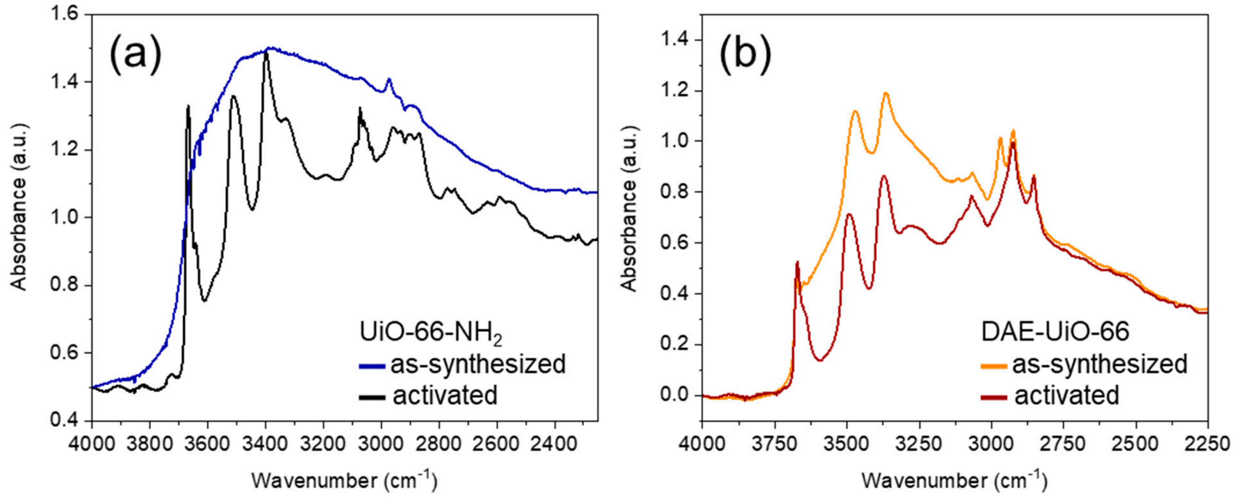

Figure S6. FTIR spectra of as-synthesized samples and those after activation: UiO-66-NH2 (**a**) and DAE-UiO-66 (**b**).

6. Stability

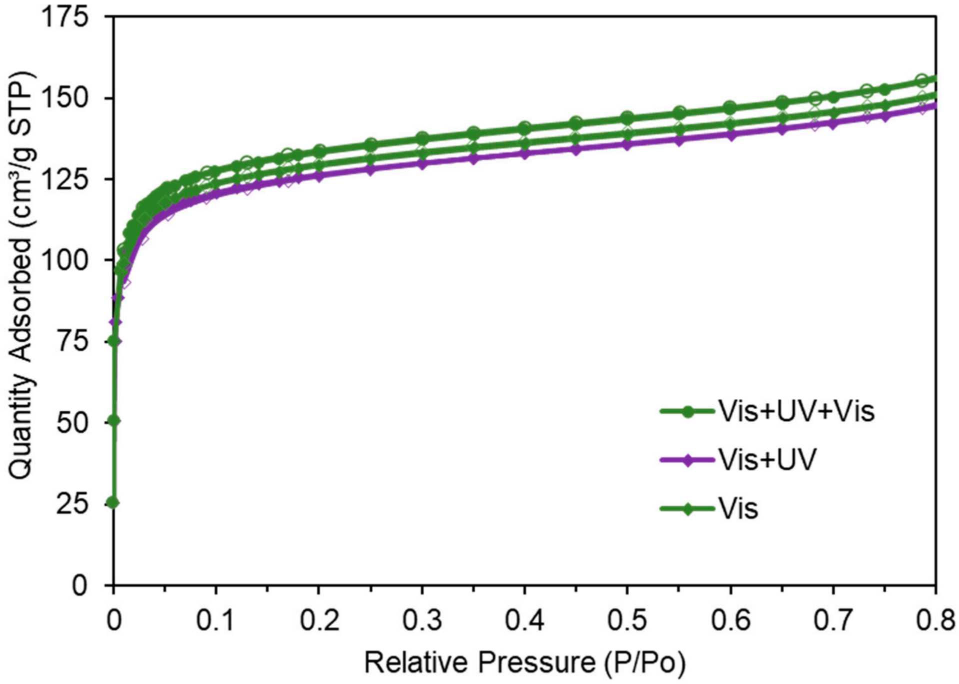

Figure S7. Nitrogen adsorption-desorption isotherms of sample DAE-UiO-66 under sequential irradiation with visible light (520 nm, green plots) and UV-light (450 nm, violet plot). Filled markers designate adsorption branches of isotherms; empty markers represent desorption ones. .

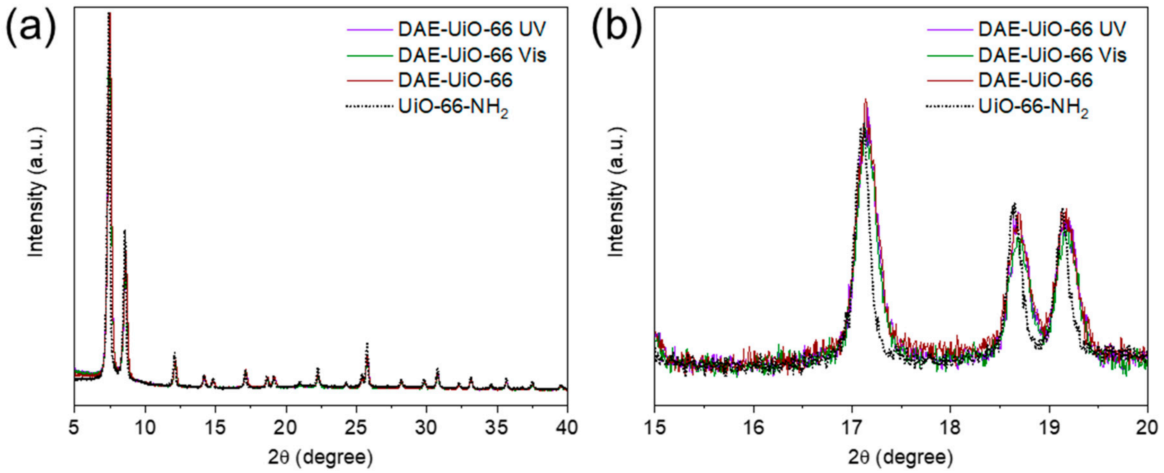

Figure S8. Powder XRD patterns of samples UiO-66-NH2 (black dotted line), DAE-IiO-66 (solid red line), DAE-UiO-66 irradiated with visible light with wavelength 520 nm (solid green line), and DAE-UiO-66 irradiated with UV-light with wavelength 450 nm after irradiation with visible light (solid violet line). Part (**a**) demonstrates all 2θ range of profiles, while part (**b**) represents the magnification of the region 15–20 degrees.
